# Endovascular treatment of a ruptured portal vein pseudoaneurysm secondary to a large stomach ulcer

**DOI:** 10.1186/s42155-025-00553-y

**Published:** 2025-05-03

**Authors:** John C. DuBois, Aaron M. Rohr, Ian T. Kozlowski, Zachary S. Collins

**Affiliations:** 1 University of Kansas School of Medicine, 3901 Rainbow Boulevard, Kansas City, KS 66103 USA; 2 The University of Kansas Health System, 3901 Rainbow Boulevard, Kansas City, KS 66103 USA

## Abstract

This case describes the endovascular treatment of a 55-year old female with a ruptured portal vein pseudoaneurysm secondary to a large stomach ulcer resulting in massive active hemorrhage into the adjacent stomach. This patient presented with persistent hypotension secondary to chronic GI blood loss and simultaneous septic shock. After an abrupt drop in blood pressure, the patient arrived in the angiography suite where the ruptured pseudoaneurysm was apparent on portal venogram. Emergent stent assisted coil embolization was performed to stabilize the patient and treat the portal vein pseudoaneurysm. To our knowledge, this case presents the only portal vein pseudoaneurysm secondary to a gastric ulcer.

**Level of Evidence** Level 4, Case-report

## Background

Portal vein aneurysms are rare. Out of all venous system aneurysms, the portal vein comprises only 3% [1]. A systematic literature search including from 2015, found that there have been only 190 reported cases of portal vein aneurysms [2] although this number has likely risen since then due to an increase in abdominal imaging. One review of portal vein aneurysms had a mean age of 54.85 years with 53% of the study population being male [3]. Portal vein aneurysms are most seen extrahepatically at the portal vein main trunk, the confluence of the splenic and mesenteric veins, the portal vein bifurcation, the branches of the portal vein, and some non-bifurcating vasculature including the splenic vein and superior mesenteric vein [3]. The etiology of portal vein aneurysms include both congenital and acquired causes [4]. Documented acquired causes include chronic liver disease, the most common cause, pancreatitis, trauma-induced, Budd-Chiari syndrome, splenomegaly in the context of thalassemia major, splenic artery aneurysm, and long-term cholelithiasis [3]. The portal vein pseudoaneurysm presented here protruded from the main portal vein and likely arose due to the adjacent 30 mm ulcer at the gastrojejunal anastomosis (previous Roux-en-Y).

On imaging, portal vein aneurysms have been demonstrated to be fusiform most commonly, but saccular and bilobular presentations have also been reported [5]. The best modality to assess size and morphology of aneurysms is a CT with contrast, but ultrasound may also be used for continuous monitoring in conservative management [5]. In the case presented here, a venogram demonstrated a 1.4 cm × 1.3 cm diverticular outpouching.

While the most common symptom reported by patients with portal vein aneurysms is abdominal pain, specific complications have been noted. These include rupture, portal hypertension, thrombosis, biliary tract compression, duodenal compression, and inferior vena cava obstruction [2]. There are two routes of management for portal vein aneurysms. Conservative management with surveillance can be done for asymptomatic patients [2]. Alternatively, surgical intervention is indicated for complicated portal vein aneurysms which include aneurysms with rupture, thrombosis, aneurysms that demonstrate an increased risk of spontaneous rupture, and patients that are symptomatic [2]. Surgical approaches include open, which can be used for portal vein aneurysms that are growing quickly or show signs of thrombosis [3]. The open approach could include an aneurysmectomy for fusiform aneurysms or an aneurysmorrhaphy for saccular aneurysms [3]. In cases where the patient suffers from portal hypertension or the patient is hemodynamically unstable, it is advantageous to treat with an endovascular approach [3]. Of patients that undergo surgery for portal vein aneurysms, some research suggests a post-operative mortality rate of 17.5% [2].

Due to the patient’s active hemorrhage and hemodynamic instability, emergent intervention was required via an endovascular approach.

## Case presentation

A 55-year-old female with a complex past medical history on chronic anticoagulation presented with primarily hemorrhagic and concomitant septic shock secondary to recurring acute GI bleed. CTA of the abdomen and pelvis was performed where contrast was noted in the excluded stomach of a previous Roux-en-Y surgery. An EGD demonstrated a large stomach ulcer measuring 30 mm at the gastrojejunal anastomosis with active hemorrhage. She underwent GelFoam embolization of the gastroduodenal artery in concert with medical management, however she demonstrated continued GI bleeding and failed resuscitation at outside facilities.

On admission to our institution, a CT of the abdomen and pelvis with contrast demonstrated a 1.5 cm portal vein pseudoaneurysm, near the portal vein confluence, likely secondary to erosion from the stomach ulcer. An exploratory surgery with portal vein reconstruction was considered but decided against due to high morbidity. A week after admission to our facility, the patient experienced an abrupt and persistent drop in blood pressure despite aggressive resuscitation. Interventional radiology was consulted for endovascular intervention.

**Fig. 1:**
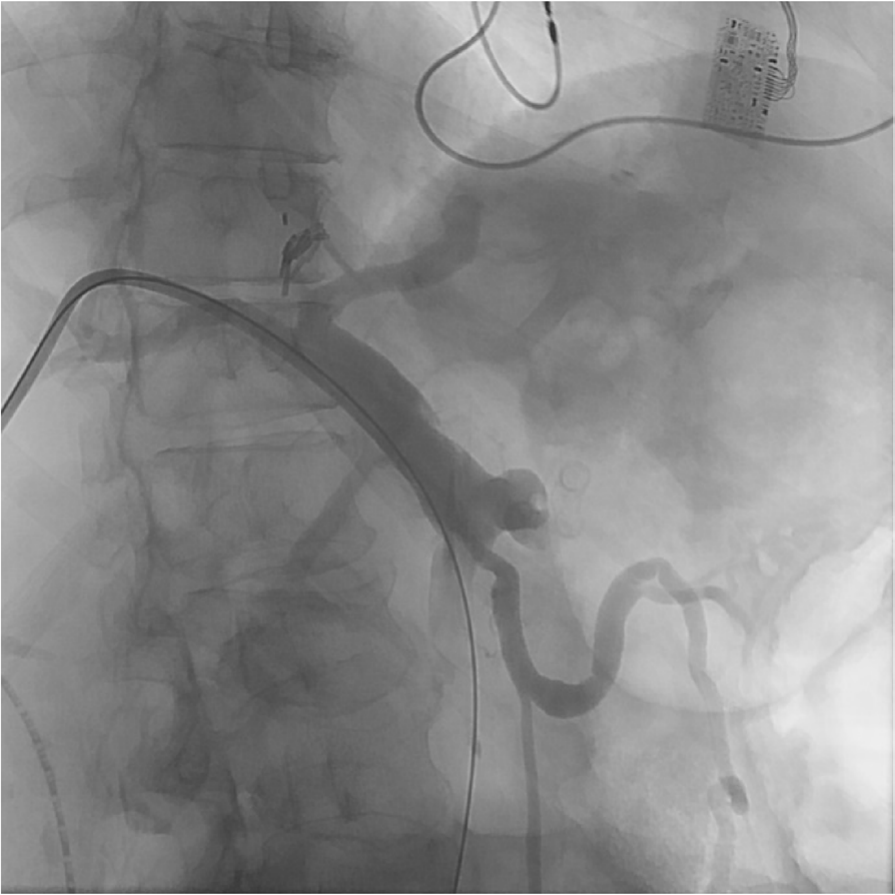
Initial portal venogram through access sheath shows patent portal system with saccular pseudoaneurysm

**Fig. 2:**
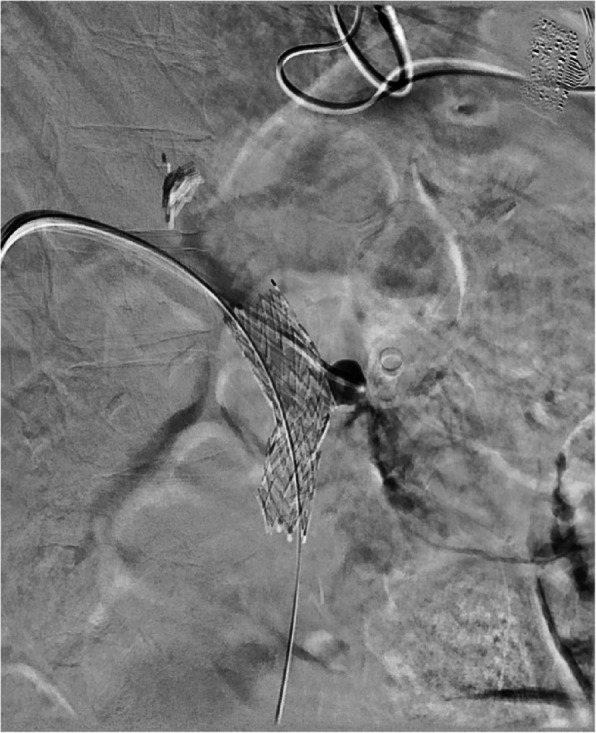
JB1 catheter in pseudoaneurysm sac showing active contrast extravasation into adjacent stomach

**Fig. 3:**
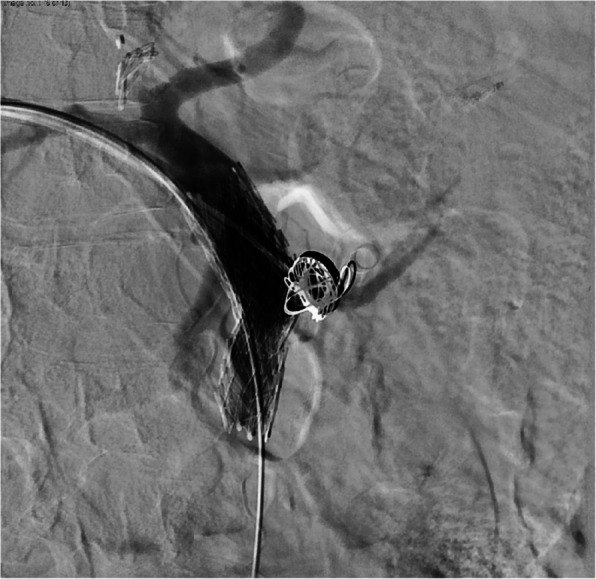
Final portal venogram through access sheath demonstrating complete hemostasis of the pseudoaneurysm sac with no extravasation. The splenic vein, SMV, and MPV remain widely patent

Upon arrival to the angiography suite, a portal venogram with intervention was planned. Ultrasound guidance was used to access the right portal vein in a transhepatic approach with a 22-gauge Chiba needle (Cook, Bloomington, IN). After the stylet was removed and contrast injected, a 0.018-inch wire (EV3, Plymouth, MN) was passed centrally into the main portal vein and the needle was exchanged for a transitional dilator. The inner stylet and wire were removed, and contrast was injected to confirm placement within the main portal vein. A 6 French vascular sheath (Cook, Bloomington, IN) was placed and a portal venogram was performed. This demonstrated a portal vein pseudoaneurysm arising from the proximal main portal vein that measured 1.4 × 1.3 cm that was actively hemorrhaging into the adjacent stomach (Fig. 1). A 20 mm balloon (Bard, Temp, AZ) was immediately placed and insufflated into the main portal superior mesenteric vein/main portal vein to temporarily tamponade the hemorrhage. A rapid response was activated, and massive transfusion protocol was initiated before proceeding given patient instability. The sheath was then upsized to a 9 French vascular sheath (Cook, Bloomington, IN). Next, an 18 mm by 80 mm fenestrated Abre (Medtronic, Minneapolis, MN) stent was placed in the SMV and main portal vein traversing the inferior mesenteric vein, splenic vein, and pseudoaneurysm origins. A Glidewire was used to select the pseudoaneurysm sac and a JB1 catheter (Angiodynamics, Latham, NY) was advanced into the sac (Fig. 2). Three coils including two 20 mm by 20 cm and one 20 mm by 30 cm endovascular coils (Terumo, Somerset, NJ) were then used to pack the pseudoaneurysm sac. Once coil embolization was complete, a vial of Obsidio (Boston Scientific, Marlborough, MA) was delivered into the pseudoaneurysm sac through a 2.8 French Progreat (Terumo, Somerset, NJ). After delivery, a portal venogram was repeated that showed complete hemostasis with no filling of the pseudoaneurysm sac (Fig. 3). After wire/catheter removal, venogram was performed that showed a widely patent main portal vein and superior mesenteric vein stent with a solid coil and conformable embolic pack in the pseudoaneurysm sac that showed no contrast filling. Hemostasis was achieved with an Amplatzer plug (Abbott Cardiovascular, Chicago, IL) within the hepatic parenchyma just beyond the portal vein puncture site. The patient left the angiography suite in critical but stable condition. The patient initially experienced improvement after this emergent life-saving intervention. Over the next two months, they endured a complicated hospital course due to unrelated comorbidities and ultimately died.

## Conclusions

This case describes a 55-year-old female with active hemorrhage from a ruptured portal vein pseudoaneurysm into an adjacent stomach. Due to the intimate association of the pseudoaneurysm at the confluence of the splenic vein and SMV, covered stent placement was not an option. The pseudoaneurysm was also broad based, making coiling alone extremely difficult and would likely lead to non-target embolization with coil dislodgement. Stent assisted coiling was the most viable option, but as the patient decompensated, a liquid embolic within the coil pack was used to achieve rapid occlusion. The case provides a unique etiology for acute hemorrhage and acute lifesaving intervention from an endovascular approach.

Two previous cases have described an approach to treating portal vein pseudoaneurysms using a stent graft [6, 7]. To utilize this approach, the pseudoaneurysm described in our case would have necessitated splenic vein and IMV coil embolization due to its location. This is a viable treatment option, but we opted to maintain as much anatomic blood flow as we could. Due to the lack of literature describing outcomes of coil embolizing splenic vein and IMV, we favored to maintain anatomic portal flow while also achieving pseudoaneurysm closure. A separate case described a portal vein pseudoaneurysm treated with a covered stent [8]. This is an ideal approach to treating portal vein pseudoaneurysms. Due to the location of the pseudoaneurysm described in our case and a desire to avoid excluding mesenteric branches, a coil pack with liquid embolization was used.

## Data Availability

N/a.

## References

[1] López-Machado E, Mallorquín-Jiménez, F., Medina-Benítez, A., Ruiz-Carazo, E., & Cubero-García, M. Aneurysms of the portal venous system: ultrasonography and CT findings. Eur J Radiol. 1998;26(2):210–4. 9518231 10.1016/s0720-048x(96)01146-1

[2] Laurenzi A, Ettorre GM, Lionetti R, Meniconi RL, Colasanti M, Vennarecci G. Portal vein aneurysm: what to know. Dig Liver Dis. 2015;47(11):918–23. 26188840 10.1016/j.dld.2015.06.003

[3] Kurtcehajic A, Zerem E, Alibegovic E, Kunosic S, Hujdurovic A, Fejzic JA. Portal vein aneurysm-etiology, multimodal imaging and current management. World Journal of Clinical Cases. 2023;11(4):725. 36818612 10.12998/wjcc.v11.i4.725 PMC9928716

[4] Fulcher A, Turner M. Aneurysms of the portal vein and superior mesenteric vein. Abdom Imaging. 1997;22:287–92. 9107652 10.1007/s002619900191

[5] Dalal PS, Raman SP, Horton KM, Fishman EK. Portal vein aneurysms: imaging manifestations and clinical significance. Emerg Radiol. 2013;20:453–7. 23700119 10.1007/s10140-013-1131-y

[6] Suzuki K, Igami T, Komada T, Mori Y, Yokoyama Y, Ebata T, Nagino M. Stent-graft treatment for extrahepatic portal vein hemorrhage after pancreaticoduodenectomy. Acta Radiologica Open. 2015;4(6):2058460115589338. 26137314 10.1177/2058460115589338 PMC4475512

[7] Oor JE, Groeneweg E, Bloemsma GC, Bokkers RP, Klaase JM. Endovascular Management of a Portal Vein Pseudoaneurysm following Pancreatoduodenectomy: A Case Report and Review of Literature. Case Rep Gastroenterol. 2025;19(1):7–13. 39981167 10.1159/000542585 PMC11666264

[8] Walton H, Yu D, Imber C, Webster G. Portal vein pseudoaneurysm secondary to pancreatic lymphoma and biliary stent insertion: a rare cause of haemobilia. CVIR endovascular. 2018;1:1–6. 30652138 10.1186/s42155-018-0011-7 PMC6319504

